# Ensembl Genomes 2018: an integrated omics infrastructure for non-vertebrate species

**DOI:** 10.1093/nar/gkx1011

**Published:** 2017-10-30

**Authors:** Paul Julian Kersey, James E Allen, Alexis Allot, Matthieu Barba, Sanjay Boddu, Bruce J Bolt, Denise Carvalho-Silva, Mikkel Christensen, Paul Davis, Christoph Grabmueller, Navin Kumar, Zicheng Liu, Thomas Maurel, Ben Moore, Mark D McDowall, Uma Maheswari, Guy Naamati, Victoria Newman, Chuang Kee Ong, Michael Paulini, Helder Pedro, Emily Perry, Matthew Russell, Helen Sparrow, Electra Tapanari, Kieron Taylor, Alessandro Vullo, Gareth Williams, Amonida Zadissia, Andrew Olson, Joshua Stein, Sharon Wei, Marcela Tello-Ruiz, Doreen Ware, Aurelien Luciani, Simon Potter, Robert D Finn, Martin Urban, Kim E Hammond-Kosack, Dan M Bolser, Nishadi De Silva, Kevin L Howe, Nicholas Langridge, Gareth Maslen, Daniel Michael Staines, Andrew Yates

**Affiliations:** The European Molecular Biology Laboratory, The European Bioinformatics Institute, The Wellcome Trust Genome Campus, Hinxton, Cambridgeshire CB10 1SD, UK; Cold Spring Harbor Laboratory, 1 Bungtown Rd, Cold Spring Harbor, NY 11724, USA; USDA-ARS NAA Plant, Soil and Nutrition Laboratory Research Unit, Cornell University, Ithaca, NY 14853, USA; Rothamsted Research, Department of Biointeractions and Crop Protection, Harpenden, Hertfordshire, AL5 2JQ, UK

## Abstract

Ensembl Genomes (http://www.ensemblgenomes.org) is an integrating resource for genome-scale data from non-vertebrate species, complementing the resources for vertebrate genomics developed in the Ensembl project (http://www.ensembl.org). Together, the two resources provide a consistent set of programmatic and interactive interfaces to a rich range of data including genome sequence, gene models, transcript sequence, genetic variation, and comparative analysis. This paper provides an update to the previous publications about the resource, with a focus on recent developments and expansions. These include the incorporation of almost 20 000 additional genome sequences and over 35 000 tracks of RNA-Seq data, which have been aligned to genomic sequence and made available for visualization. Other advances since 2015 include the release of the database in Resource Description Framework (RDF) format, a large increase in community-derived curation, a new high-performance protein sequence search, additional cross-references, improved annotation of non-protein-coding genes, and the launch of pre-release and archival sites. Collectively, these changes are part of a continuing response to the increasing quantity of publicly-available genome-scale data, and the consequent need to archive, integrate, annotate and disseminate these using automated, scalable methods.

## OVERVIEW AND ACCESS

Ensembl Genomes (http://www.ensemblgenomes.org) is organised as five sites, each focused on one of the traditional kingdoms of life: bacteria, protists, fungi, plants and (invertebrate) metazoa. Vertebrate metazoa are the focus of the Ensembl project (1); Ensembl Genomes provides a complementary set of interfaces for non-vertebrate species. Our goals are to provide high-quality reference genome sequence and annotation for every species for which these are available; to represent genomic diversity for all species of major research interest; to link out to phenotypic data and resources containing biological material; and to provide a set of tools that allows users to interrogate these data in conjunction with their own. This paper describes the current state of the resource and ongoing progress towards these aims.

For all species included in the resource, we currently provide access to genome sequence and annotations of protein-coding and non-coding genes. Transcriptional, genetic variation, and comparative analysis data are additionally available for many species. For most species, these data are automatically imported using standard pipelines from the archives of the International Nucleotide Sequence Database Consortium (INSDC), i.e. the European Nucleotide Archive (2), GenBank (3), and the DNA Database of Japan (4), the European Variation Archive (http://www.ebi.ac.uk/eva), Wikipedia, and other open access sources. For a few species of particular research or socio-economic importance, additional high-value data sets are identified and manually imported. A link to ‘information and statistics’ for each genome provides information including the methods of assembly, references to publications and archival submission numbers, and the date the assembly was first incorporated into the resource.

Interactive access to all data is provided through a web interface providing genome browsing capabilities: users can scroll through a graphical representation of a DNA molecule at various levels of resolution, seeing the relative locations of features—including conceptual annotations (e.g. genes, SNP loci), sequence patterns (e.g. repeats) and experimental data (e.g. expressed RNA sequences mapped onto the genome) that supports the primary annotations. Functional information is provided through direct curation, import from the UniProt Knowledgebase (5), or imputation from protein sequence (using the classification tool InterProScan (6)). Various tools for text and sequence search, data upload and data analysis are available, allowing researchers to examine their own data in the context of the reference sequence and annotation.

Ensembl data have traditionally been stored in a set of MySQL databases which can be directly accessed via a public MySQL server (host: mysql.ebi.ac.uk port: 4157 username: anonymous) and additionally through well-developed Perl and RESTful APIs that provide an object-oriented framework for working with genomic data. Increasingly the Ensembl web application directly utilizes data files stored in archival resources (such as the European Nucleotide archive), avoiding the need for database builds and improving the speed of response. All data in the resource is open-access, and both database dumps and common data sets (e.g. DNA, RNA and protein sequence sets and sequence alignments) can be directly downloaded in bulk via FTP (ftp://ftp.ensemblgenomes.org).

Ensembl Genomes data is also organised in additional databases, constructed using the BioMart data warehousing system (7), optimised around common gene- and variant-centric queries. The BioMart framework provides web-based query building tools, and a variety of other interfaces for interactive and programmatic access. BioMarts are not currently available for Ensembl Bacteria.

Ensembl Genomes is updated 4–5 times a year in synchrony with updates to Ensembl, utilising the same software as the corresponding Ensembl release. The overall suite of Ensembl Genomes interfaces mirrors those provided for vertebrate genomes in Ensembl, allowing users to access genomic data from across the tree of life in a consistent manner. In addition, Ensembl Genomes contributes to collaborative database projects focused on various domains of life, including Gramene (http://www.gramene.org) (8) for plants, PhytoPath (http://phytopathdb.org) (9) for plant pathogens, VectorBase (http://www.vectorbase.org) (10) for invertebrate vectors of human pathogens, and WormBase (http://www.wormbase.org) (11) for helminths. In these projects, we work with our partners to develop common datasets, which are made available through both Ensembl Genomes and additional project-specific interfaces.

## NEW AND IMPROVED GENOME ASSEMBLIES

Ensembl Genomes has continued to grow in 2016 and 2017 (see Table 1). The resource contains all annotated assemblies from fungal and protist species that are present in the INSDC, and it is planned to extend this approach to plants and metazoa within the next year. However, owing to the very large number of bacterial genome assemblies now available, a filter has been applied from release 35 onwards to exclude new genome assemblies which fail to add significant diversity to the overall collection. This approach mirrors that already adopted by the UniProt Knowledgebase for filtering data from bacterial genomes (12).

**Table 1.** Growth in Ensembl Genomes 2015–2017

Release version	Date	Number of genomes				
		Number in brackets indicates genomes directly imported from INSDC.				
		Ensembl Bacteria	Ensembl Protists	Ensembl Fungi	Ensembl Plants	Ensembl Metazoa
28	August 2015	23 001 (23,001)	133 (101)	407 (359)	41 (8)	55 (1)
37	September 2017	44 048 (44,048)	189 (157)	811 (760)	45 (12)	68 (2)
Increase		21 047 (21,047)	56 (56)	404 (401)	4 (4)	13 (1)

Species of particular societal, research or taxonomic interest that have been recently incorporated include *Bombus impatiens* (the common bumblebee) (13), *Octopus bimaculoides* (the California two-spot octopus) (14), *Sarcoptes scabiei* (the itch mite, the cause of scabies) (15), *Beta vulgaris* (sugar beet) (16) and *Brassica napus* (rapeseed) (17). Several existing assemblies have also been upgraded, and a number of previously highly fragmented genomes have now been incorporated in more contiguous forms. Cereal genomes are of particular interest, owing to their large size (at 16 Gb the polyploid bread wheat genome is the largest genome currently represented in the resource) and complex repeat structure, which have historically made them difficult to assemble. However, recent advances in technology are now yielding dramatically improved genome assemblies even for cereals. For example, the latest assembly of the barley genome (18) has been added to the resource and comprises just 6,347 scaffolds with an N50 of 1.9 Mb (cf. the previous assembly, which contained 376 261 unscaffolded contigs of over 1 Kb in length with an N50 of just 1.4 Kb). While this might not yet be a complete molecular assembly, it is closer to a finished state than many smaller genomes in the resource that were sequenced and assembled using previous technologies. Similarly, the bread wheat genome is also undergoing rapid improvement. A significantly improved new assembly, the TGAC1.0 assembly (19), has already been incorporated in the resource, and we are currently working on a further upgrade to incorporate the IWGSC RefSeq v1.0 assembly (currently available at https://www.wheatgenome.org).

## INCREASED DATA FROM COMMUNITY ANNOTATORS

Through our involvement in the VectorBase project, we are able to provide community-provided gene models (as modifications or extensions to the previous genome-wide annotation) for 26 genomes. Community members can access an instance of Apollo (20), an online genome editing tool, to assess evidence, and submit proposed changes, which are quickly visible in the browser and which are subsequently assessed for inclusion in a revised gene set. We have subsequently expanded our support for community annotation to enable the complete re-annotation of two fungal phytopathogen species, *Botrytis cinerea* (21) and *Blumeria graminis*, by members of their respective communities, and have incorporated the revised gene sets within Ensembl Fungi. We are currently working with the *Zymoseptoria tritici* community in a similar initiative, and are exploring ways of providing generic access to Apollo for all species in future.

## INTEGRATION OF RNA-SEQ DATA

Relatively recently, transcriptional evidence for gene models was scarce for many non-model species. Today, data from many thousands of RNA-Seq experiments are present in the nucleotide sequence archives; however, the raw read sequence is not immediately useful. We have therefore developed a pipeline to automatically identify sequence read data in the INSDC archives and align them to the corresponding genomic sequence. These alignments are stored in Compressed Read Alignment Map (CRAM) format (22) and are resubmitted to the ENA for persistent archiving. Data from technical replicates are merged by default. To make these thousands of tracks accessible in Ensembl Genomes, alignments derived from a single experiment (‘Study’ in the ENA data model) are organised in track hubs (23), a convenient format that can group sets of related positional data prior to their visualisation as tracks in a genome browser. To date, alignments have been generated for plants, invertebrate vectors and plant pathogens, and will shortly be produced for other fungi, protists, and metazoan species. A summary of currently available alignments is shown in Table 2.

**Table 2.** RNA-seq alignment tracks by division

Division	Tracks	Experiments	Species
Protists	71	36	3
Fungi	6384	4822	24
Plants	29 836	1418	43
Metazoa	198	105	34

**Figure 1.**
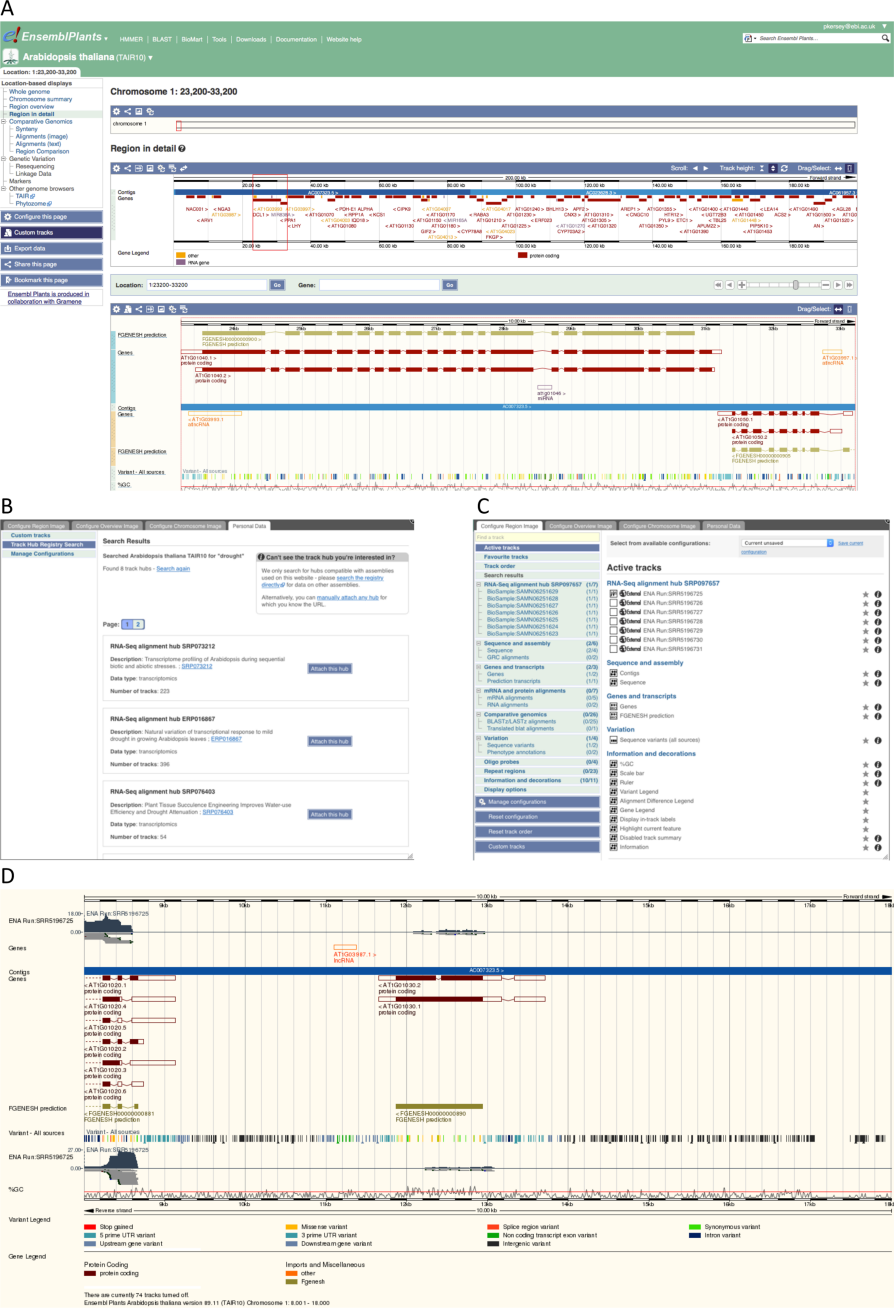
Data discovery and visualisation of alignment data in Ensembl Plants. While browsing the genome, a user can follow the ‘Custom tracks’ option in the left-hand menu (panel **A**). This gives access to a menu that searches the track hub registry for hubs that are anchored on this genome and match a specified metadata search term (panel **B**). Having selected a hub, the user is led through to a second menu where the tracks contained in the hub can be figured for display (panel **C**). When the configuration is complete, the selected tracks appear in the browser as selected (panel **D**).

Track hubs are stored and indexed in a dedicated registry (http://trackhubregistry.org), and a search interface over this registry has been implemented in the Ensembl browser. Users of the browser can directly identify hubs containing data located on the genome they are currently browsing, filter the list to select only those hubs whose annotated meta data matches a given search term, and then select tracks from within the chosen hub for visualization. This process is illustrated in Figure 1. Researchers can also submit their own track hubs directly to the registry, and thereby expose their data through Ensembl Genomes and other track-hub compliant browsers.

## FAST PROTEIN SEQUENCE SEARCH WITH HMMER

**Figure 2.**
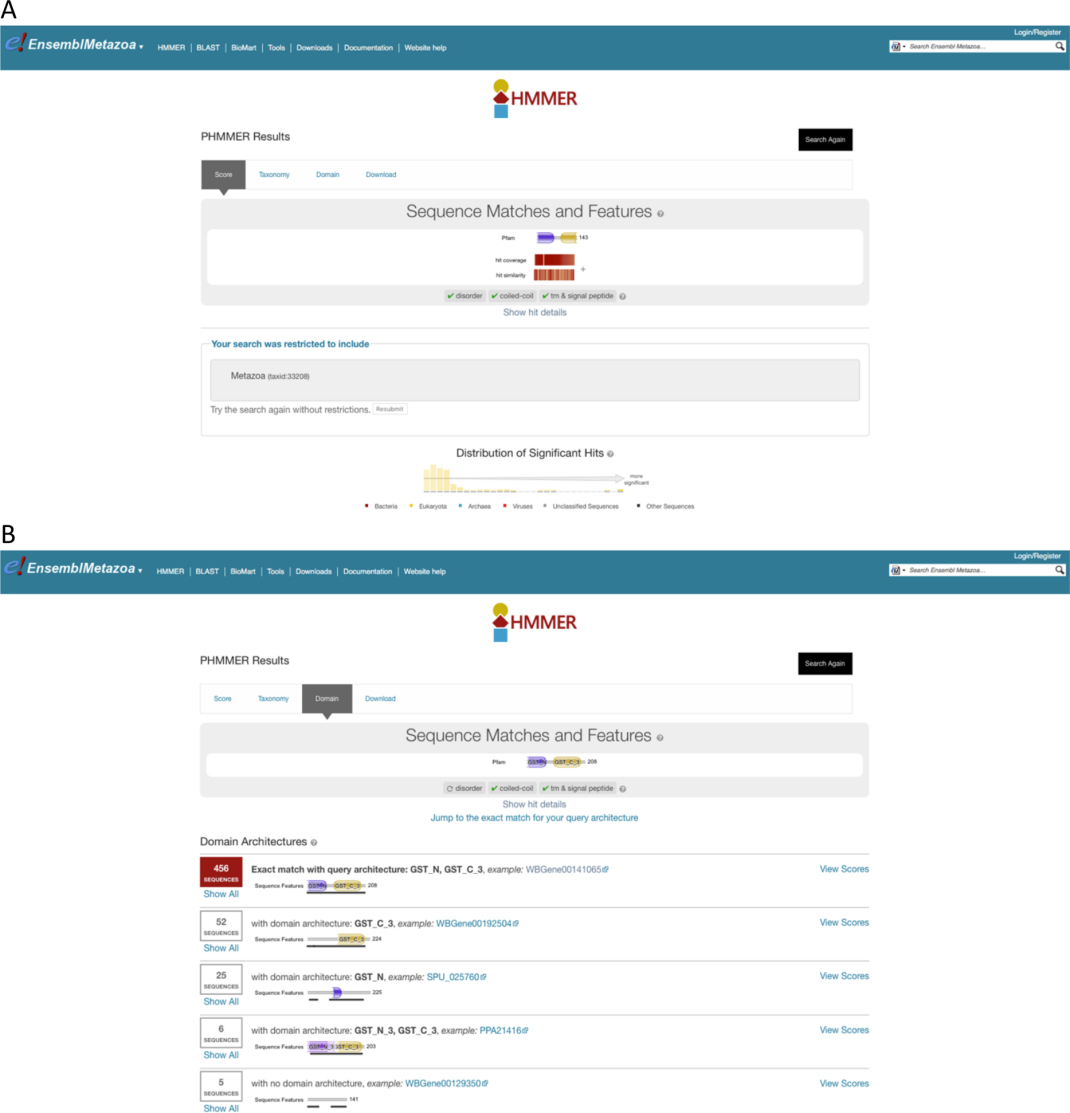
Hidden Markov Model search integrated into Ensembl Genomes. Results from a protein search using Hidden Markov Models, implemented using HMMer3, in Ensembl Genomes. Various options are available in a tabbed display including (i) a description of the domain architecture of the query sequence, and a graphical summary of the distribution of the significance scores of matches against the library (panel **A**) (ii) a breakdown of matching library sequences ordered by domain architecture (panel **B**).

A new fast, accurate protein sequence search has been introduced, utilising the HMMER3 tool (24), which uses Hidden Markov Models to find matching sequences. The search has been implemented by indexing Ensembl Genomes protein sequences within an existing public HMMER3 server (25), and connecting this server to pages for the entry of query sequence and the visualisation of results within the Ensembl Genomes site. After a search has completed, users are shown a taxonomic breakdown of significant hits, a presentation of the alignments of query and target sequences, and a view of the domain architecture of the top hit (see Figure 2). BLAST search (26) of both protein and nucleotide sequences continues to be available.

## PRE-RELEASE SITES AND ARCHIVE SITES

On publication of an updated version of a genome assembly of an important species, we use pre(-release) sites to make the initial data quickly available before we have had time to recompute the full range of analyses performed on previous version. The previous version remains available in the normal site until the newer version is fully described. Genomes are removed from the pre-site when analysis is complete and the new assembly is ready to migrate to the main site. Pre-sites are accessed at URLs such as http://pre.metazoa.ensembl.org, and are advertised prominently within the main site when available. Assemblies recently made available on pre-sites include new assemblies for *Beauveria bassiana* (a parasite of arthropods), *Fusarium graminarium* and *Fusarium culmorum* (both plant pathogens), *Hordeum vulgare* (barley), *Triticum aestivum* (bread wheat), and the diatom *Phaeodactylum tricornutum*, all of which have subsequently migrated to the live site.

While improved assemblies are obviously desirable, they can be problematic for researchers currently attempting to complete a lengthy data analysis, and the loss of previous versions from the website also makes it harder for scientists to check on results previously published. Moreover, genome alignment tracks lose utility if the reference sequence used is no longer available to view in a browser. Ideally, older assemblies would remain available in the browser, even after new versions have been created. As a first step towards achieving this, we have made available an archived version of release 32 of Ensembl Plants, alongside the live version. It is planned to shortly deploy archival versions of all Ensembl Genomes sites containing the release 37 data set, and thereafter to supplement these with approximately annual updates.

## OTHER IMPROVEMENTS

Annotation of non-coding RNA genes is often poor or non-existent in archival submissions. An updated pipeline has been written for the identification of non-coding RNA genes, using updated versions of Rfam (27) and tRNAscan-SE (28) and improved filtering of the results, and has been applied to 162 eukaryotic genomes, resulting in an additional 213 717 gene annotations (an average of ∼1300 per genome). These are accessible alongside protein-coding annotations in the database downloads and browser.

We have improved our integration with PHI-base (29), a database of genes involved in plant pathogenesis, using sequence similarity to locate genes not linked to the genome. The number of cross-referenced genes has increased from 1491 to 2756, which comprises 98.9% of the potentially mappable genes. Plant genes have been linked to pathways in the Plant Reactome (http://plantreactome.gramene.org) (30) database.

Finally, Ensembl Genomes is now available for download in RDF format.

## FUTURE PERSPECTIVES: FISHING IN THE DATA DELUGE

For some years, as genome sequencing technology has continued to improve, it has been forecast every organism of interest would soon have a completed genome sequence. Yet while the quantity of published sequence has steadily increased, the best assembly available for many species has continued to be highly fragmented (and indeed, many recent genome assemblies have been more fragmented than those produced with earlier technologies). However, the availability of new assemblies for wheat and barley, and the increasing availability of unbroken whole chromosome assemblies for smaller genomes (e.g. many fungal species), indicates that the era of universal reference genome sequences is finally dawning. Since Ensembl Genomes organises data around contiguous sequences, the challenge of data presentation is simplified as assemblies become more complete; in addition, more contiguous assemblies are likely to better represent repeat structure, heterozygosity, and other phenomena that can lead to a mis-interpretation of the true genomic content of an organism.

Nonetheless, Ensembl Genomes faces various challenges as the total quantity of available data continues to rise. Firstly, it becomes increasingly important that access to data is provided computationally as well as via interactive interfaces. Ensembl and Ensembl Genomes have always provided a variety of data downloads and APIs for this purpose, and the availability of data in RDF format represents a further offering in this respect. Secondly, data processing pipelines need to be sufficiently automatic and performant to be able to process the available volume of data. The implementation of procedures for the automatic import of reference genomes from the public archives (whose use will be expended within the next year to cover invertebrate metazoa and plant species), and for the automatic generation of tracks from alignment data, have already enabled a massive increase in the quantity of data contained within the resource. A priority for the near-future is the establishment of a pipeline to allow for the automatic representation of any variant call data represented in the European Variation Archive (http://www.ebi.ac.uk/eva) within the framework of reference annotation/interpretation through Ensembl interfaces. This model is dependent, of course, on data producers continuing to subscribe to long-established norms about submitting assembly and annotation data to the INSDC databases, and other data types to appropriate broad-scope repositories. If data is archived in universal archives, it becomes easier for resources such as Ensembl Genomes to integrate and interpret them; the more dispersed data is, the higher the overheads of re-use. In our opinion, it is important that the norms of archival submission are maintained, and we try to practice what we preach: when Ensembl Genomes generates alignment data, these are submitted back into the ENA and advertised through the Track Hub registry, and thus made available in any compliant browser outside of the Ensembl infrastructure. A culture of data sharing improves all resources, and thereby empowers researchers.

The third challenge, in an environment of data plenitude, is to allow users to discover and select data of interest to visualise or analyse. The grouping of tracks into track hubs, and the provision of interfaces by which hubs can be discovered according to their metadata and selectively imported into the Ensembl framework, is a scalable model for data discovery and selection.

The usefulness of this model is critically dependent on the quality of metadata with which the data has been annotated, including the correct identification of the species and strain to which the data set belongs, and descriptions of the aims of the overall experiment and the differences between individual tracks. However, there are a number of obstacles to the acquisition of such metadata: experiments are diverse and designing standards for describing them are consequently difficult; retro-fitting meta data to independently submitted archival submissions is an innately costly process; the most scalable solutions therefore require that data is annotated with metadata prior to submission to the public archives, but data generators may be poorly incentivised to do so, inexpert in the relevant data standards, and actively hostile to being asked to supply the same information more than once. Finding a solution to these problems requires community acceptance of appropriate standards, the development of helpful tools for data validation and submission, and the automatic re-use of metadata between different resources. We are currently working on a project to further develop existing metadata standards (31) for the plant domain, and to capture submitted metadata to link information in Ensembl Genomes (for example, genotype data for individual crop cultivars) to external repositories holding phenotypic data and/or physical stocks. The BioSamples database (32) will be used to connect different repositories containing data derived from related materials. A similar approach is likely needed across the taxonomic space to ensure that specific archived data can be discovered, visualised and used in Ensembl tools and elsewhere.
